# Retraction: MiR26a and miR144 inhibit proliferation and metastasis of esophageal squamous cell cancer by inhibiting cyclooxygenase2

**DOI:** 10.18632/oncotarget.28890

**Published:** 2026-05-20

**Authors:** Ying Shao, Peng Li, Sheng-Tao Zhu, Ji-Ping Yue, Xiao-Jun Ji, Dan Ma, Li Wang, Yong-Jun Wang, Ye Zong, Yong-Dong Wu, Shu-Tian Zhang

**Affiliations:** ^1^Department of Gastroenterology, Beijing Friendship Hospital, Capital Medical University, National Clinical Research Center for Digestive Disease, Beijing Key Laboratory for Precancerous Lesion of Digestive Diseases, Beijing, China; ^2^Department of Critical Care Medicine, Beijing Friendship Hospital, Capital Medical University, Beijing, China

**This article has been retracted:** Oncotarget has concluded its investigation of this article, and identified several instances of image reuse and overlap:

- Figure 3A: Morphological images of EC9706, EC109, and EC109-vector cells were reused from Figure 2A of 2015 publication of the same group [1].
- Figure 3B: the migration and invasion transwell assay images of the control cells EC9706 and EC9706-vector, EC109 and EC109-vector were reused from Figure 4A of reference [1].
- Figures 3B/4E: There is an overlap between the EC9706 migration and invasion images. Additionally, the EC9706-mir26a invasion image in Figure 3B was reused in Figure 4E as the EC9706-mir144 invasion image.

The authors did not respond to requests for comment or provide the original data. Consequently, the editorial decision has been made to retract this paper. Oncotarget has reached out to all authors to notify them of this retraction but has received no response.

Original article: Oncotarget. 2016; 7:15173–15186. 15173-15186. https://doi.org/10.18632/oncotarget.7908
